# Bioconversion of Alpha-Cembratriene-4,6-diol into High-Value Compound Farnesal Through Employment of a Novel *Stenotrophomonas maltophilia* H3-1 Strain

**DOI:** 10.3390/molecules30051090

**Published:** 2025-02-27

**Authors:** Shen Huang, Jiaming Cheng, Huibo Hu, Aamir Rasool, Robina Manzoor, Duobin Mao

**Affiliations:** 1 College of Food and Biological Engineering, Zhengzhou University of Light Industry, Zhengzhou 450002, China; huangshen@zzuli.edu.cn (S.H.); cjm7768@163.com (J.C.); 18939361913@163.com (H.H.); 2 Institute of Biochemistry, University of Balochistan, Quetta 87300, Pakistan; rasool.amir@gmail.com; 3 Department of Biotechnology and Bioinformatics, Lasbela University of Agriculture, Water and Marine Sciences, Uthal 90150, Pakistan; rubina.manzoor86@gmail.com

**Keywords:** *Stenotrophomonas*, bioconversion, α-cembratriene-4,6-diol, farnesal, α-CBT-diol

## Abstract

Alpha-cembratriene-4,6-diol (α-CBT-diol) is a complex diterpenoid primarily found in *Solanaceae* (i.e., tobacco leaves), *Pinaceae*, and marine corals. Due to its intricate chemical structure, it serves as a precursor for several aroma compounds, including farnesal. Farnesal and its derivatives have applications across various fields, such as the fragrance and flavor industry, pharmaceuticals, agriculture, and cosmetics. In this study, *Stenotrophomonas maltophilia* H3-1, a strain capable of efficiently biodegrading α-CBT-diol into farnesal, was isolated from soil and identified through 16S rDNA sequence analysis. *S. maltophilia* H3-1 biodegraded 93.3% of α-CBT-diol (300 mg/L) within 36 h when grown under optimized culture conditions, including a temperature of 40 °C, pH of 8, 2 g/L maltose, and 2 g/L ammonium sulfate. Theoretically, this strain can produce 201 mg/L of farnesal during the biotransformation of α-CBT-diol. The putative α-CBT-diol bioconversion pathway expressed in *S. maltophilia* H3-1 is also proposed. This is the first study to report the bioconversion of α-CBT-diol into the high-value compound farnesal using a novel *S. maltophilia* H3-1 strain. It highlights that other compounds found in tobacco can also be bioconverted into valuable products.

## 1. Introduction

Cembranoids are a class of naturally occurring diterpenoids that are commonly found in various plant families such as *Solanaceae* (e.g., *Nicotiana tabacum*) and *Pinaceae* [1], marine corals [2,3] (Li et al. 2006), and some animals [4]. Over 300 cembranoid compounds have been identified from various natural sources, with α-CBT-diol being the most abundant in nature [5]. Arnarp et al. utilized *Tripterygium wilfordii* cells harvested from different growth stages as biocatalysts to explore the bioconversion products of α-CBT-diol. Their research revealed that α-CBT-diol can produce various products due to its susceptibility to epoxidation and hydroxylation at the C-10, C-12, and C-13 positions [6].

Farnesal is an FDA-approved flavoring agent and adjuvant used in forming farnesal-loaded pH-sensitive polymeric micelles to prevent and treat dental caries [7]. Farnesal is a precursor of farnesol [8,9], a bioactive compound used in various industries, including food, cosmetics, and perfumes [10,11,12]. More specifically, farnesol demonstrates antimicrobial, anti-tumor, cardioprotective, hepatoprotective, and neuroprotective properties (in Alzheimer’s and Parkinson’s diseases) [13,14,15,16,17,18]. Furthermore, farnesol has also been shown to be active against allergic asthma, diabetes, atherosclerosis, obesity, and hyperlipidemia [19,20,21].

In this study, the *S. maltophilia* H3-1 strain, which efficiently converts α-CBT-diol from tobacco to farnesal, was isolated, characterized, and described from the soil. Subsequently, the fermentation conditions were optimized for the efficient conversion of α-CBT-diol into farnesal and the optimal growth of the *S. maltophilia* H3-1 strain. The cellular localization of the α-CBT-diol-degrading enzymes expressed in *S. maltophilia* H3-1 was also determined, and a mechanism for converting α-CBT-diol into farnesal was proposed. To our knowledge, this is the first study to report the bioconversion of α-CBT-diol into farnesal through the use of a microbe.

## 2. Results and Discussion

### 2.1. Isolation, Identification, and Characterization of α-CBT-diol Biodegrading Bacteria

**Figure 1:**
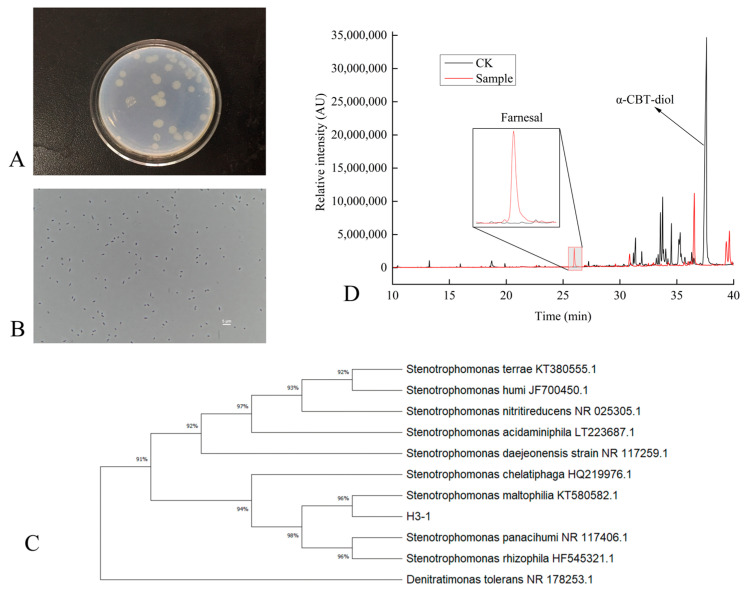
(**A**–**D**) Characterization of *S. maltophilia* H3-1 strain. (**A**) colony morphology of *S. maltophilia* H3-1 strain; (**B**) cell morphology of *S. maltophilia* H3-1 strain; (**C**) phylogenetic tree showing the evolutionary relationship of *S. maltophilia* H3-1 strain; and (**D**) GC-MS chromatogram showing the production of farnesal from α-CBT-diol by *S. maltophilia* H3-1 strain.

This study systematically isolated and purified a single *S. maltophilia* H3-1 strain colony from the soil. The morphological characterization revealed that colonies of the *S. maltophilia* H3-1 strain on LB medium were light yellow and round, with a bright surface and raised center (Figure 1A). This type of appearance of the colonies is consistent with previous studies that describe the typical morphology of *S. maltophilia* colonies on LB medium [22]. This appearance is linked to the production of extracellular enzymes and bioactive compounds, contributing to the strain’s adaptability to environmental conditions [23]. Gram staining showed that the *S. maltophilia* H3-1 strain cells were thin rods, flagella-less, and had a size of 0.2–0.3 × 0.7–1.0 μm (Figure 1B). The lack of flagella indicates a reduced need for motility in its native soil environment. This suggests that biofilm formation is an alternative mode of adaptation, a common trait among *Stenotrophomonas* species [24].

To identify and classify the bacterial strain isolated from the soil, we sequenced its 16S rDNA, which was 1497 bp in length. The sequence was then compared with previously sequenced bacterial 16S rDNAs using BLAST (2.14.0) homology. The results showed that the 16S rDNA had high similarity with *S. maltophilia* KT580582T (99.8%), *Stenotrophomonas chelatiphaga* HQ219979T (99.8%), and *Stenotrophomonas acidaminiphila* LT223687T (99.8%). The high sequence similarity with the strains above suggests that our isolated strain belongs to the *Stenotrophomonas* genus. This genus is known for its broad range of metabolic capabilities, which allows it to adapt to various environmental conditions [25,26].

A phylogenetic tree based on the 16S rDNA sequences of the isolated strain was constructed. The tree showed that the H3-1 strain formed a distinct phyletic line with *S. maltophilia* and is located on a separate branch (Figure 1C). The placement of *S. maltophilia* H3-1 on a separate branch represents its distinct lineage while indicating a close evolutionary relationship with *S. maltophilia* strains [27,28].

Initially, the ability of *S. maltophilia* H3-1 to biodegrade α-CBT-diol into farnesal was determined by growing it in a selective medium containing α-CBT-diol as the sole carbon source. The conversion of α-CBT-diol to farnesal was confirmed by the compound retention index (RI) and GC-MS analysis (Figure 1D). The successful conversion of CBT-diol to farnesal by *S. maltophilia* H3-1 highlights its potential ability to transform complex organic compounds [29].

### 2.2. Growth and α-CBT-diol Bioconversion Curves of S. maltophilia H3-1 Strain

**Figure 2:**
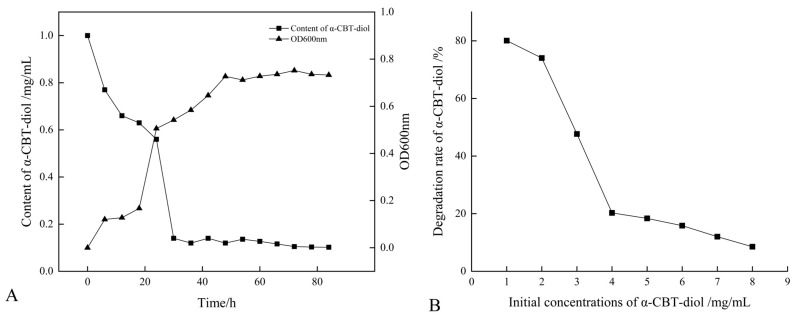
(**A**) Relationship between the growth curve and α-CBT-diol bioconversion curve of *S. maltophilia* H3-1 strain; and (**B**) relationship between initial concentrations of α-CBT-diol and bioconversion capacity of *S. maltophilia* H3-1 strain.

The growth curve of the *S. maltophilia* H3-1 strain was constructed by recording the optical density at OD_600_nm. The optical data show that the H3-1 strain grew slower during the first 20 h of the growth period, but after that, it rapidly started growing and consuming a large amount of α-CBT-diol (Figure 2A). *S. maltophilia* H3-1 strain started consuming α-CBT-diol (non-natural carbon source) for growth due to the selective pressure. It achieved growth optima at the 36th hour, which indicates that the number of cells producing and decaying were in equilibrium (Figure 2A). Moreover, it also shows that the H3-1 strain took 36 h to adapt to consuming α-CBT-diol as a carbon source for growth. Consequently, the bioconversion of α-CBT-diol became visible at the 36th hour of the growth period of the *S. maltophilia* H3-1 strain.

The extended lag phase represents the period during which the *S. maltophilia* H3-1 strain adjusted to the novel carbon source [30], demonstrating its potential applications in bioremediation.

### 2.3. Optimization of Growth Parameters and Concentration of α-CBT-diol

*S. maltophilia* H3-1 strain demonstrated a high α-CBT-diol bioconversion rate; however, an increase in the initial concentration of α-CBT-diol led to a corresponding decrease in the bioconversion capacity of the H3-1 strain (Figure 2B). The results of this study show that the optimum concentration of α-CBT-diol is 1 mg/mL. A concentration higher than this negatively affected the strain’s bioconversion capacity (Figure 2B).

This finding suggests that the *S. maltophilia* H3-1 strain efficiently biodegrades α-CBT-diol into farnesal but is sensitive to high substrate concentrations, which may have saturated the enzymes responsible for degrading α-CBT-diol. This accumulation could have resulted in reduced growth and an overall decrease in the bioconversion rate [31].

**Figure 3:**
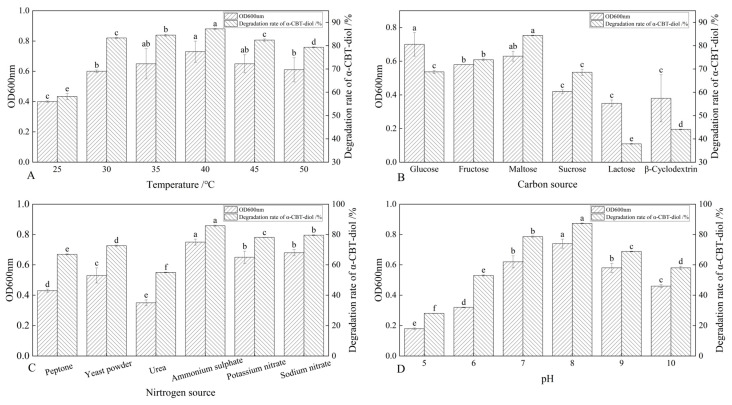
(**A**–**D**) Optimization of growth parameters and their effect on the bioconversion rate of α-CBT-diol and the biomass production of *S. maltophilia* H3-1 strain: optimization of temperature (**A**), optimization of carbon sources (**B**), optimization of nitrogen sources (**C**), and optimization of pH of fermentation medium (**D**).

The *S. maltophilia* H3-1 strain was cultured at different temperatures, including 25 °C, 30 °C, 35 °C, 40 °C, 45 °C, and 50 °C, to determine the optimal temperature at which this strain demonstrates the highest α-CBT-diol bioconversion rate. The results showed that the *S. maltophilia* H3-1 strain degraded α-CBT-diol at the lowest rate (~57.6%) at 25 °C, but the bioconversion rate increased reciprocally with the temperature rise. The optimal temperature was 40 °C, at which *S. maltophilia* H3-1 degraded ~88.7% of α-CBT-diol in the growth medium and accumulated the maximum amount of biomass, reflected in an OD_600_ value of 0.73. As the temperature increases, the kinetic energy of molecules rises, enhancing enzyme–substrate interactions and thereby increasing reaction rates [32,33]. However, the α-CBT-diol bioconversion rates decreased to 83.1% and 78.3%, respectively, when *S. maltophilia* H3-1 was grown at 45 °C and 50 °C (Figure 3A). The enzymes are prone to denaturation when their thermal stability is exceeded, causing a loss of their three-dimensional structure and catalytic function. In addition to enzyme denaturation, excessive heat can disrupt cell membranes and other critical cellular components, impairing overall metabolic function [32,33]. *S. maltophilia* H3-1 strain is not thermophilic; therefore, temperatures above 40 °C could inflict damaging effects on the enzymatic machinery responsible for the bioconversion of α-CBT-diol into farnesal and the heat-labile cellular components of this microorganism. Consequently, we selected 40 °C as the optimum temperature for further fermentation experiments.

**Figure 4:**
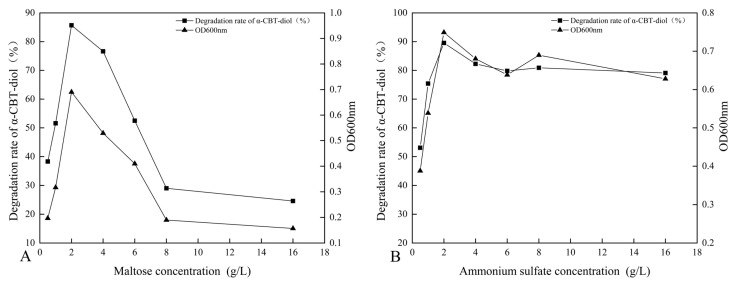
(**A**,**B**) Dose optimization of maltose and ammonium sulfate and their effect on the bioconversion rate of α-CBT-diol and the biomass production of *S. maltophilia* H3-1 strain: optimization of maltose concentration (**A**), optimization of ammonium sulfate (**B**).

The synergistic effect of different carbon sources, including glucose, fructose, maltose, sucrose, lactose, and β-Cyclodextrin, on the bioconversion rate of α-CBT-diol was determined by growing the *S. maltophilia* H3-1 strain in growth media, each containing one of these carbon sources (1 g/L,). The tested carbon sources demonstrated a synergistic effect on the bioconversion rate of α-CBT-diol in the following order: maltose > fructose > glucose > sucrose > β-Cyclodextrin > lactose (Figure 3B). Maltose exerted the most substantial positive synergistic effect, achieving an 86.8% α-CBT-diol bioconversion rate when *S. maltophilia* H3-1 was grown in a growth medium containing 2 g/L maltose as a complementary carbon source (Figure 4A). The optical density, representing biomass accumulation, was 0.65, higher than other carbon sources except for glucose. Although the *S. maltophilia* H3-1 strain exhibited higher growth in the medium containing glucose, it demonstrated a lower α-CBT-diol bioconversion rate (Figure 3B). The findings suggest that the choice of carbon source is pivotal for maximizing the efficiency of *S. maltophilia* H3-1 in degrading α-CBT-diol. The positive synergistic effect of maltose may be attributed to its ability to enhance the expression of specific catabolic enzymes or improve the strain’s overall metabolic activity in utilizing this carbon source [34]. Future studies could further explore the mechanisms behind the enzymatic pathways activated by different carbon sources to enhance the bioconversion processes of α-CBT-diol. The α-CBT-diol bioconversion rate and optical density decreased when maltose concentrations higher than 2 g/L were used to grow *S. maltophilia* H3-1. However, as the concentration continued to increase beyond 2 g/L, both the bioconversion rate and OD_600_ value declined sharply (Figure 4A). This reduction was likely due to the high maltose concentration causing increased osmotic pressure in the medium, which inhibited bacterial growth and the synthesis of degradative enzymes [35].

The effect of organic and inorganic nitrogen sources, such as ammonium sulfate, sodium nitrate, potassium nitrate, yeast extract, peptone, and urea, on the bioconversion rate of α-CBT-diol was evaluated by growing the *S. maltophilia* H3-1 strain in a medium containing one of the nitrogen sources separately at a concentration of 1 g/L. It was observed that inorganic nitrogen sources had a significantly stronger stimulating effect on the rate of α-CBT-diol bioconversion compared to organic nitrogen sources (Figure 3C). Notably, the use of 2 g/L ammonium sulfate as the sole nitrogen source in the fermentation medium strongly promoted the bioconversion rate of α-CBT-diol and biomass production (OD_600_), recorded at 86.5% and 0.74, respectively (Figure 4B). The order of the effect of nitrogen sources on the bioconversion rate of α-CBT-diol was as follows: ammonium sulfate > sodium nitrate > potassium nitrate > yeast powder > peptone > urea. (Figure 3C) However, the addition of urea to the growth medium as the nitrogen source demonstrated the worst effect on the bioconversion rate of α-CBT-diol (56.5%) and biomass production (0.35). Ammonium sulfate significantly enhanced the bioconversion rate of α-CBT-diol by the *S. maltophilia* H3-1 strain. This is likely due to the rapid release and assimilation of ammonium ions compared to other nitrogen sources tested in this study, which are readily available for microbial metabolism and promote the enzyme production necessary for the bioconversion of α-CBT-diol [36]. When the *S. maltophilia* H3-1 strain was grown in a medium containing ammonium sulfate higher than 2g/L, it did not further enhance the optical density and bioconversion rate of α-CBT-diol (Figure 4B). Overall, the use of ammonium sulfate as a nitrogen source demonstrated a positive impact on the growth and bioconversion of α-CBT-diol by the *S. maltophilia* H3-1 strain.

The pH of the fermentation medium plays a crucial role in generating the high-yield production of the desired product; therefore, we also studied the effect of different pH values on the bioconversion rate of α-CBT-diol and biomass production. Our results showed that the *S. maltophilia* H3-1 strain grew very slowly in an acidic growth medium. The bioconversion rate of α-CBT-diol and biomass production increased proportionally with a rise in the pH value of the fermentation medium. We observed the maximum bioconversion rate of α-CBT-diol (87.5%) and biomass production (0.73) at pH 8. However, both the bioconversion rate and biomass production were severely affected when the pH exceeded 8 in the fermentation medium. This indicates that an overly alkaline pH is not suitable for the bioconversion of α-CBT-diol and the growth of the *S. maltophilia* H3-1 strain. Therefore, we selected pH 8 as the optimal initial pH of the fermentation medium (Figure 3D). It is well established that pH influences the ionization state of the medium, which can directly affect the solubility and availability of nutrients as well as the structural stability of enzymes involved in bioconversion [37]. The slower growth observed at acidic pH levels suggests that the *S. maltophilia* H3-1 strain may be less efficient at regulating internal pH, leading to a reduced metabolic rate and lower enzyme production [38]. The proportional increase in the bioconversion rate and biomass production with rising pH values reflects the optimal enzyme activity in a slightly alkaline environment, as seen with pH 8. This is consistent with other studies showing that many microbial strains exhibit higher metabolic activity under mildly alkaline conditions [39,40]. However, the detrimental effect of pH values higher than 8 may be attributed to enzyme denaturation or the disruption of cellular processes due to excess alkalinity, which can impair the overall bioconversion process.

The optimal liquid culture conditions for *S. maltophilia* H3-1 were determined as follows: a bioconversion time of 36 h, a temperature of 40 °C, a pH of 8, 2 g/L maltose as the carbon source, and 2 g/L ammonium sulfate as the nitrogen source, based on the bioconversion rate of α-CBT-diol. Under these conditions, the bioconversion rate of α-CBT-diol reached 93.3%, resulting in a theoretical farnesal production of 201 mg/L, assuming a 1:1 molar conversion. The results indicate that the strain exhibited its highest bioconversion efficiency under these optimized growth conditions.

### 2.4. Confirmation of Membrane Association of Enzymes Catalyzing the Conversion of α-CBT-diol into Farnesal

**Figure 5:**
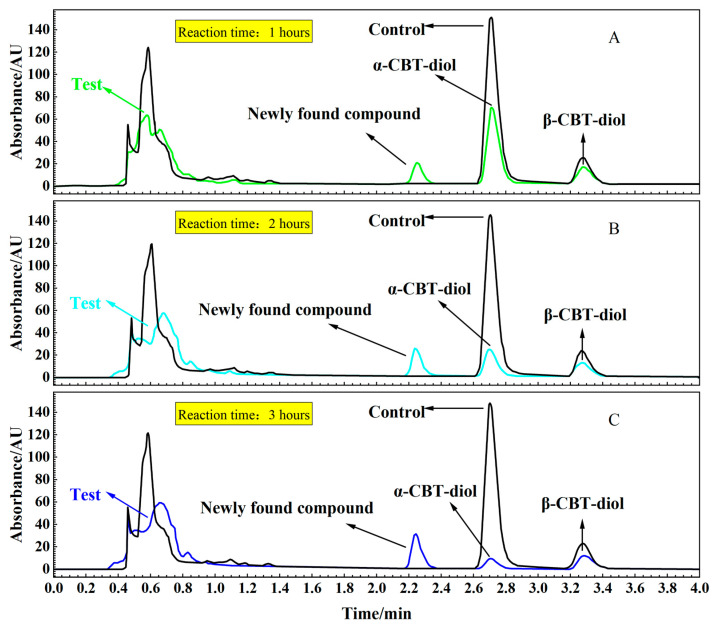
(**A**–**C**). HPLC analysis of the supernatant from reaction mixtures: Time-course analysis of the bioconversion of α-CBT-diol into farnesal using a crude enzyme solution and α-CBT-diol (Test) (**A**: 1 h; **B**: 2 h; **C**: 3 h). Control reaction without crude enzyme solution.

To confirm the localization of the enzymes catalyzing the conversion of α-CBT-diol into farnesal, reaction mixtures were prepared with α-CBT-diol and the cytosolic and membrane components of *S. maltophilia* H3-1. Figure 5A,B, and c illustrate that α-CBT-diol gradually degraded over time and nearly completed bioconversion after approximately 3 h in the test tube experiment. The bioconversion was accompanied by forming a newly found compound, indicating the enzymatic breakdown of α-CBT-diol. Enzymatic activity was detected exclusively in the membrane component, while no bioconversion activity was observed in the cytosolic fraction. This suggests that the enzymes responsible for α-CBT-diol bioconversion are associated with the plasma membrane.

This localization of the enzymes to the membrane is consistent with many other microbial bioconversion systems, where membrane-bound enzymes often catalyze the bioconversion of hydrophobic or membrane-associated compounds. The membrane environment likely facilitates the accessibility of α-CBT-diol, which could interact more efficiently with the enzymes due to its lipid-like structure [41,42].

**Figure 6:**
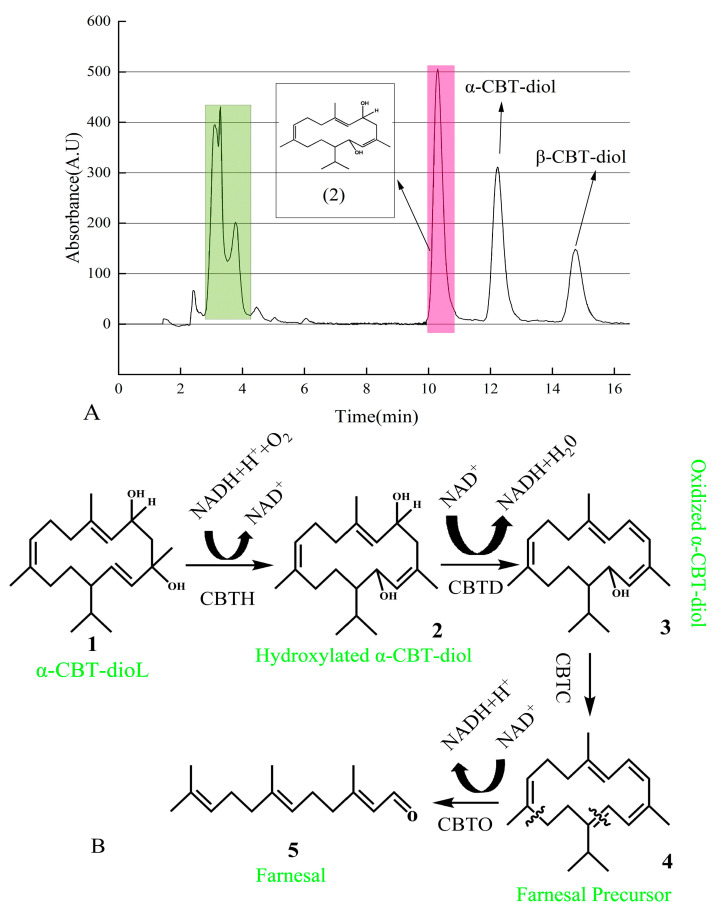
(**A**) Isolation of newly found compound using HPLC. Green peaks represent a mixture of the compounds produced during the biotransformation of α-CBT-diol. In contrast, the pink peak represents the hydroxylated α-CBT-diol, the precursor of farnesal produced during the biotransformation of α-CBT-diol. (**B**) Mechanism of α-CBT-diol bioconversion into farnesal. CBT-hydroxylase (CBTH), dehydratase (CBTD), aldehyde synthase (CBTC), and oxidoreductase catalyzes (CBTO).

The newly found compound was isolated using UPLC and identified using GC-MS (Supplementary Figure S1). After vacuum concentration and freeze-drying, a white powdery substance was obtained. The structure of this newly found compound was determined using nuclear magnetic resonance (NMR) analysis (Supplementary Figure S2a,b). As shown in Figure 6A, the pink-shadowed peak represents the newly found compound produced from α-CBT-diol.

In this study, we predicted that the bioconversion pathway of α-CBT-diol to farnesal involves a series of enzymatic reactions. In the first step, CBT-hydroxylase, likely a monooxygenase encoded by the cbtA gene, catalyzes the hydroxylation of α-CBT-diol, producing hydroxylated α-CBT-diol. This step introduces a hydroxyl group to the substrate, increasing its hydrophilicity, and consumes NADH/NADPH as an electron donor to activate molecular oxygen. In the second step, dehydratase, encoded by the cbtB gene, catalyzes the removal of a water molecule (dehydration) and an oxidation reaction, forming oxidized α-CBT-diol, a compound with a conjugated double-bond system. This step reduces NAD⁺ to NADH, contributing to the cofactor balance.

The third step involves aldehyde synthase (encoded by cbtC), which processes the oxidized intermediate through a cleavage reaction to generate farnesal precursor, a compound containing an aldehyde group. This step does not directly involve the consumption or production of NADH. In the final step, oxidoreductase, encoded by the cbtD gene, catalyzes the oxidation of the farnesal precursor to yield farnesal, a high-value aromatic aldehyde. This step also reduces NAD⁺ to NADH.

Overall, the pathway consumes one molecule of NADH/NADPH during hydroxylation (Step 1) and produces two molecules of NADH during oxidation reactions (Steps 2 and 4), effectively driving the conversion of α-CBT-diol into farnesal. This pathway highlights the enzymatic efficiency and cofactor utilization in transforming a complex diterpenoid into a commercially valuable compound (Figure 6B).

## 3. Materials and Methods

### 3.1. Isolation of *S. maltophilia* H3-1 Strain from Soil

*S. maltophilia* H3-1 strain was isolated from soil (at a depth of 3–7 cm) collected from Sanmenxia City, Henan Province, China. We weighed 10 g of the soil sample, shook it on an ultra-clean workbench to break it up, and then added it to a triangular flask containing 100 mL of sterile water. Afterward, a 1:10 sample suspension was prepared by soaking it overnight. Then, using a sterilized pipette, 1 mL of the soil sample was transferred into 100 mL of Czapek–Dox medium {(K_2_HPO_4_ (1.00 g/L), MgSO_4_·7H_2_O (0.50 g/L), KCl (0.50 g/L), NaNO_3_ (3.00 g/L), FeSO_4_·7H_2_O (0.01 g/L), sucrose (30.00 g/L)}. The Czapek–Dox medium containing the soil sample was incubated at 30 °C in a rotary incubator set at 150 rpm for 24 h to allow bacterial growth.

The *S. maltophilia* H3-1 strain was subsequently isolated by adding 1 mL of the bacterial culture obtained from the Czapek–Dox medium into 100 mL of selective medium {(MgSO_4_·7H_2_O (0.500 g/L), FeSO_4_·7H_2_O (0.005 g/L), NaCl (0.500 g/L), KH_2_PO_4_ (0.650 g/L), K_2_HPO_4_ (1.000 g/L), MnSO_4_ (0.001 g/L), (NH_4_)_2_·SO_4_ (0.500 g/L), Na_2_MoO_4_·2H_2_O (0.005 g/L), CaCl_2_·2H_2_O (0.100 g/L)} containing α-CBT-diol (0.3 g/L) and incubated at 30 °C in a rotary incubator set at 150 rpm for 2 days.

This study used the fermentation medium (selective medium with α-CBT-diol) without the bacterial inoculum as a blank. The bacterial strain, which degraded the α-CBT-diol, was identified by monitoring the change in the color of the fermentation media and analyzing the sample by GC-MS. The 1 mL culture of the α-CBT-diol-degrading bacterial strain was inoculated in the sterilized LB medium {(peptone (10 g/L), NaCl (10 g/L), yeast powder (5 g/L)}, which was then incubated at 30 °C in a rotary incubator set at 150 rpm for 24 h. The bacterial culture was then serially diluted in 10^−2^, 10^−4^, and 10^−^⁶ dilutions and spread on plates of separation medium {(selective medium with α-CBT-diol (0.3 g/L) and ager (20 g/L)} in triplicate and incubated at 30 °C for 2 days. The single colonies with a well-defined shape and transparent zones were selected for further purification by repeated streaking until no mixed colonies were visible on the plate.

The single colonies were then re-screened using the separation medium. The colonies with clear transparent zones were inoculated into 100 mL of fermentation medium incubated at 30 °C in a rotary incubator set at 150 rpm for 2 days. GC-MS detected the bioconversion products of α-CBT-diol. We analyzed the product’s structure using NMR to verify the resulting product. The isolated *S. maltophilia* H3-1 strain was then stored in 60% (*w*/*v*) glycerol (glycerol to strain ratio of 1:1) and preserved at −80 °C. All inoculations were performed on an ultra-clean workbench.

### 3.2. Morphological and Phylogenetic Classification of S. maltophilia H3-1 Strain

The cell morphology of the *S. maltophilia* H3-1 strain was analyzed and characterized using a confocal microscope (LEIKA ICC50, Leica Microsystems, Wetzlar, Germany). The gram staining was performed to classify the *S. maltophilia* strain H3-1 as a Gram-negative bacterium.

The genomic DNA of *S. maltophilia* strain H3-1 was isolated after culturing the strain in an LB medium for 12 h. The bacterial genomic DNA isolation kit (Norgen Biotek Corp, Thorold, ON, Canada) was used as per the manufacturer’s protocol.

The primers (forward) 5′-CAGAGTTTGATCCTGGCT-3′ and (reverse) 5′-AGGAGGTGATCCAGCCGCA-3′ were used to amplify the 16s rDNA using genomic DNA as a template. Shanghai Bioengineering Co., Ltd., Shanghai, China, performed the sequencing of PCR products. The 16S rDNA sequences of *Stenotrophomonas* genus strains were downloaded from NCBI for the phylogenetic analysis of the bacterial strain isolated in this study. The BLAST was used for sequence comparison, and the maximum likelihood method was employed to construct phylogenetic trees using MEGA11. The 16S rDNA sequence of *S. maltophilia* strain H3-1 was submitted with accession number QQ588742.

### 3.3. Optimization of Growth Parameters of S. maltophilia H3-1 Strain

To achieve the optimal growth and efficient bioconversion of α-CBT-diol, several growth parameters were optimized, including (i) the initial concentration of α-CBT-diol, (ii) the fermentation time, (iii) the fermentation temperature, (iv) the carbon source and content, (v) the nitrogen source and content, and (vi) the initial pH of the fermentation medium to support the growth of *S. maltophilia* strain H3-1.

The optimum fermentation time was determined by sampling the culture of *S. maltophilia* strain H3-1 grown in a fermentation medium at different times: 0 h, 6 h, 12 h, 18 h, 24 h, 30 h, 36 h, 42 h, 48 h, 54 h, 60 h, 66 h, 72 h, 78 h and 84 h. The rotation speed of the rotary incubators for all experiments was set at 150 rpm, and the temperature was maintained at 30 °C, except during the temperature optimization experiment.

The OD₆₀₀ was measured using a UV-1700 UV-Vis Spectrophotometer (Shanghai Macylab Instrument Co. Shanghai, China). A sterile fermentation medium was used as a blank control, and the measurements were repeated three times.

Subsequently, we optimized the following growth parameters: the initial concentration of α-CBT-diol (1 mg/mL, 2 mg/mL, 3 mg/mL, 4 mg/mL, 5 mg/mL, 6 mg/mL, 7 mg/mL, 8 mg/mL); temperature (25 °C, 30 °C, 35 °C, 40 °C, 45 °C and 50 °C); carbon source (glucose, fructose, maltose, sucrose, lactose β-Cyclodextrin); carbon source concentration (0.5 g/L, 1 g/L, 2 g/L, 4 g/L, 6 g/L, 8 g/L, 16 g/L); nitrogen source (ammonium sulfate, sodium nitrate, potassium nitrate, yeast powder, peptone, urea); nitrogen source concentration (0.5 g/L, 1 g/L, 2 g/L, 4 g/L, 6 g/L, 8 g/L, 16 g/L); and initial pH (5, 6, 7, 8, 9, 10).

### 3.4. Evaluate the Cellular Localization of Enzymes Catalyzing Bioconversion of α-CBT-diol into Farnesal

A 1% inoculum of *S. maltophilia* strain H3-1 was added to 100 mL of LB medium and grown for 24 h in a rotary incubator set at 150 rpm and 30 °C. The cell pellet was obtained by centrifuging the cell culture at 12,857× *g* for 20 min at 4 °C and was washed three times with BPS buffer (pH 7). The cells were disrupted using a sonicator, which was operated with a 2 s ON and 4 s OFF cycle for 15 min. The cell suspension temperature was maintained at 4 °C, and 66.7% power was applied.

The cytosolic and membrane components, including the plasma membrane, were separated by centrifuging the suspension at 12,857× *g* for 10 min at 4 °C. The presence of the enzymes capable of degrading α-CBT-diol was determined by mixing 0.3 g/L of α-CBT-diol with the cytosolic and membrane components dissolved in 5 mL of PBS. The reaction mixtures were incubated separately for 4 h in a shaking incubator set at 150 rpm and 30 °C. Samples of 1.5 mL were taken at 1 h, 2 h, and 3 h and centrifuged at 12,857× *g* at 4 °C for 5 min. The supernatants were collected and filtered using a 0.22 μm filter for ultra-high-performance liquid chromatography (UPLC) analysis to monitor the bioconversion reaction.

Chromatographic separation was performed on an ACQUITY UPLC BEH C18 column (2.1 mm × 100 mm; 1.7 μm particle size) using an ACQUITY UPLC System with 2D LC Technology (Waters, Milford, MA, USA) equipped with a UV-Vis detector. The mobile phase consisted of acetonitrile (solvent A) and ultrapure water (solvent B), following a gradient program: 0–10 min, 70% A; 6–11 min, 100% A; and 11–16 min, 80% A/20% B. The flow rate was set at 0.3 mL/min, and the column temperature was maintained at 35 °C.

The bioconversion product of α-CBT-diol was identified and confirmed by GC-MS.

### 3.5. Determination of Bioconversion Rate of α-CBT-diol

The bioconversion rate of α-CBT-diol was determined by preparing a starter culture of *S. maltophilia* strain H3-1. A 0.5% inoculum of this strain was grown in 100 mL LB medium for 24 h. Following this, 1% of the starter culture was transferred to a 100 mL fermentation medium and incubated at 30 °C in a rotary incubator set at 150 rpm. The experiment was conducted in triplicate. At various time intervals (6 h, 12 h, 18 h, 24 h, 30 h, 36 h, 42 h, 48 h, 54 h, 60 h, 66 h, and 72 h), the optical density (OD) at 600 nm was recorded, and the remaining culture was centrifuged at 12,857× *g*. The bioconversion product (farnesal) of α-CBT-diol and non-degraded α-CBT-diol were extracted from the supernatant using dichloromethane. The content of non-degraded α-CBT-diol was detected and quantified using GC-MS.

The bioconversion rate was calculated using the following formula:

$$Bioconversion\;rate\;of\;\alpha -CBT-diol=\frac{Decreased\;concentration\;of\;\alpha -CBT-diol\;}{Original\;concentration\;of\;\alpha -CBT-diol}\times 100\%$$

### 3.6. Extraction and Detection of Bioconversion of α-CBT-diol to Farnesal

The *S. maltophilia* strain H3-1 was grown in 100 mL of fermentation medium for 48 h and centrifuged at 12,857× *g* for 20 min. The supernatant was used to extract the bioconversion product, farnesal, from α-CBT-diol using 100 mL of CH_2_Cl_2_. The supernatant and CH_2_Cl_2_ were mixed evenly for 10 min using a vortex, and this process was repeated five times. The organic phase was collected and dried using a rotary evaporator [43].

An HP-5 MS column (5% phenyl methyl silox, dimensions: 30 m × 0.25 mm × 0.25 μm) was used with helium gas as the carrier at a 1 mL/min flow rate in the GC-MS. The GC-MS program was designed as follows: the temperature was raised from 50 °C to 200 °C at a rate of 5 °C/min and then increased to 260 °C at a rate of 3 °C/min. The inlet temperature was set to 280 °C, with a split ratio 3:1, and the sample volume was 1 μL.

The GC-MS (Agilent 6890 a/5975 c, Agilent Technologies Inc., Santa Clara, CA, USA) was operated in full scan mode. The MS conditions were as follows: the transmission line temperature was 280 °C; the ion source temperature was 280 °C; the quadrupole temperature was 150 °C; the ionization mode was electron ionization (EI) with an electron energy of 70 eV; the solvent delay time was 8 min; and the scanning range (*m*/*z*) was 35–550 u. The 99.98% pure standard of farnesal was used for detection and confirmation.

## 4. Conclusions

In this study, *S. maltophilia* H3-1 was successfully isolated from soil and identified through 16S rDNA sequencing. The strain demonstrated high efficiency in the bioconversion of α-CBT-diol into farnesal, a valuable compound widely used in the fragrance, flavor, pharmaceutical, and agricultural industries. By optimizing growth conditions such as the temperature, pH, and carbon and nitrogen sources, we achieved an α-CBT-diol bioconversion rate of 93.27% within 36 h at a temperature of 40 °C, a pH of 8, and using maltose and ammonium sulfate, which resulted in the production of farnesal. The enzymes catalyzing the bioconversion of α-CBT-diol into farnesal were found to be localized to the plasma membrane of the *S. maltophilia* H3-1 strain, with no activity detected in the cytosolic components.

## Data Availability

Data are contained within the article.

## Supplementary Materials

The following supporting information can be downloaded at: https://www.mdpi.com/article/10.3390/molecules30051090/s1. Supplementary Figure S1: The MS spectrum of Farnesal. Supplementary Figure S2a,b: Confirmation of structure of farnesal using NMR spectroscopy.
