# Supplementary material for: Bioconversion of Alpha-Cembratriene-4,6-diol into High-Value Compound Farnesal Through Employment of a Novel Stenotrophomonas maltophilia H3-1 Strain
Source: Molecules. 2025 Feb 27;30(5):1090. doi: 10.3390/molecules30051090 (PMC11901948; doi:10.3390/molecules30051090)
Supplement: Supplementary file 1 [file molecules-30-01090-s001.zip › molecules-3422511-supplementary.pdf]

Bioconversion of Alpha-cembratriene-4,6-diol into High-Value Compound Farnesal through Employment of a Novel *Stenotrophomonas maltophilia* H3-1 Strain

by Shen Huang1， Jiaming Cheng1， Aamir Rasool2, Huibo Hu1, Robina Manzoor3 and Duobin Mao1,*

Supplementary Figure S1: The MS spectrum of Farnesal.

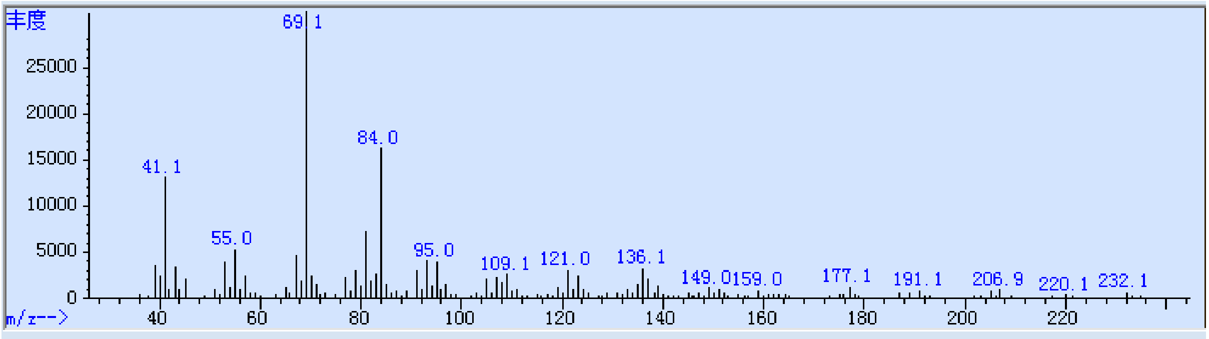

Supplementary Figure S2a,b: Confirmation of structure of farnesal using NMR spectroscopy

A:

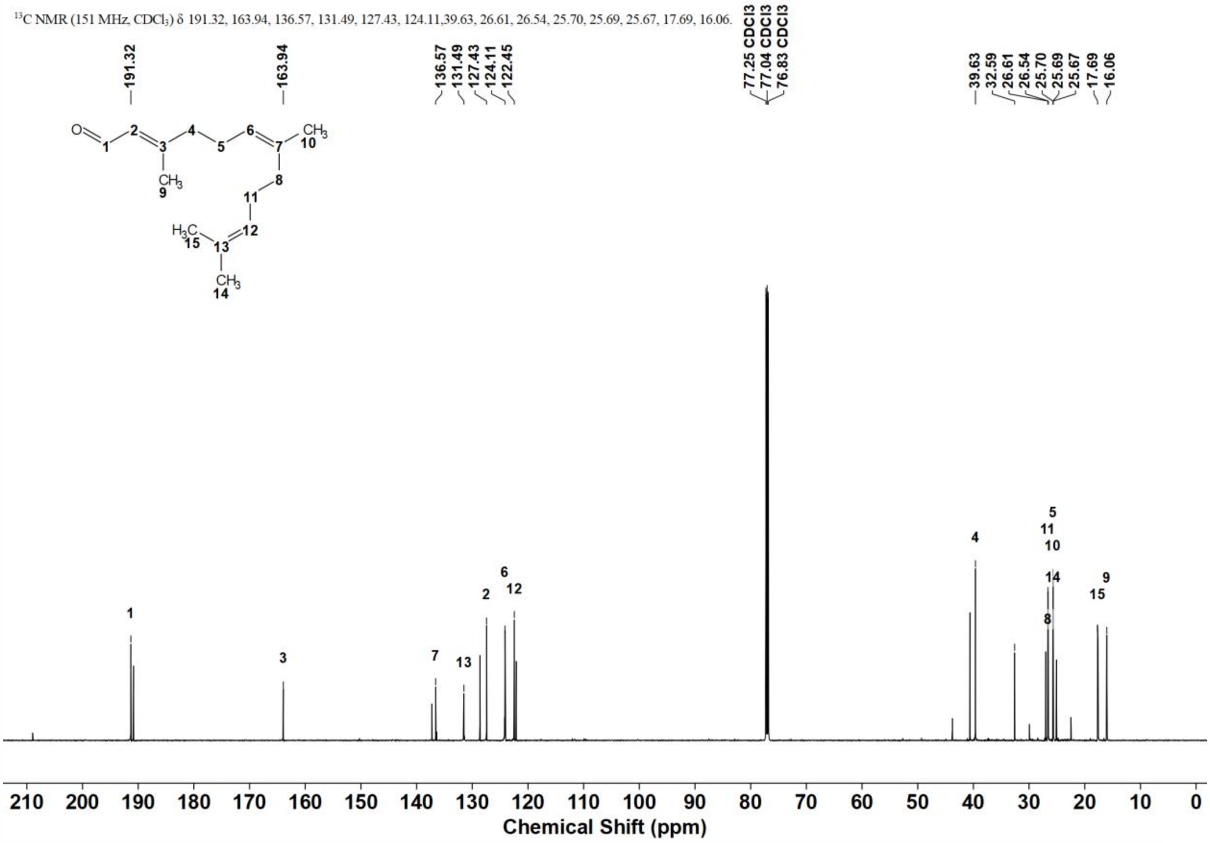

Figure. S1 ^{13}CNMR of farnesal

13C NMR (151 MHz, Chloroform-d) δ 191.32, 163.94, 136.57, 131.49, 127.43, 124.11, 122.45, 39.63, 26.61, 26.54, 25.70, 25.69, 25.67, 17.69, 16.06.

B

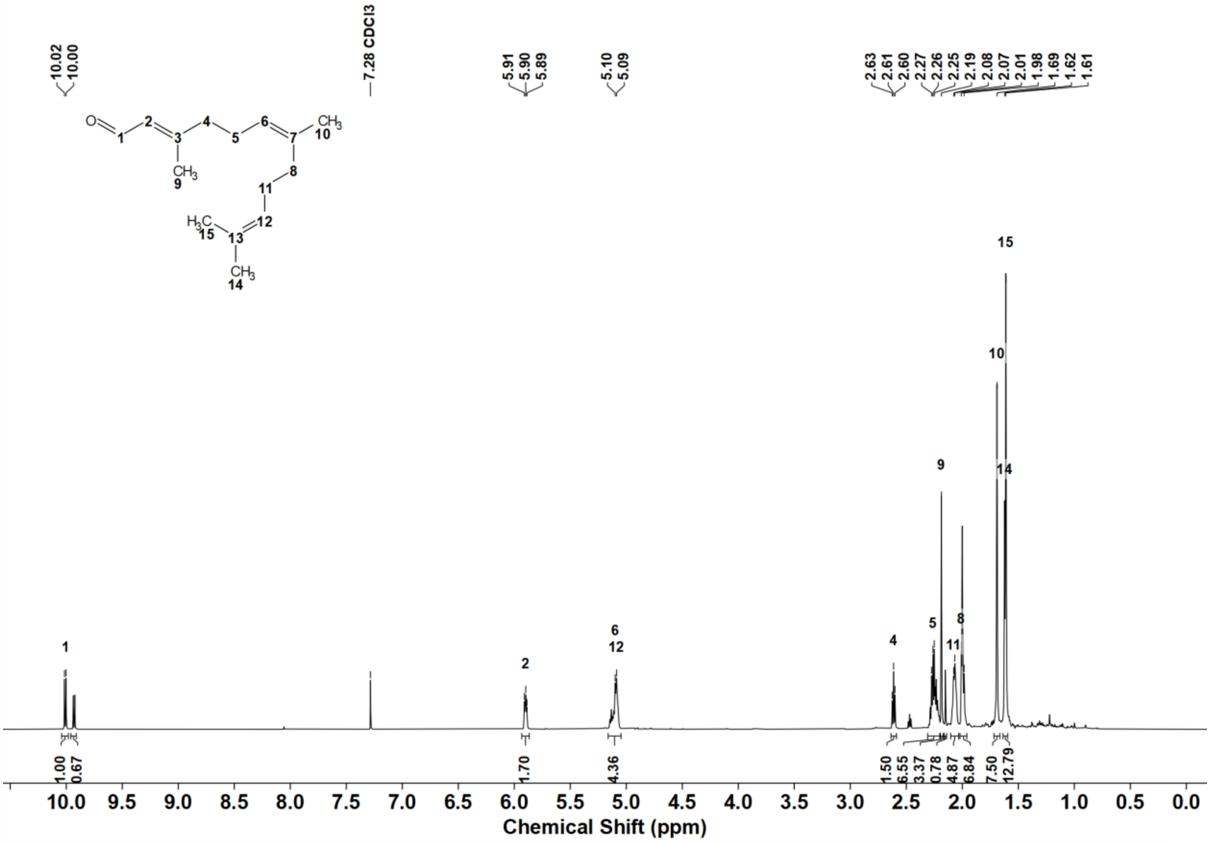

Figure. S2 ^{1}HNMR of farnesal

1H NMR (600 MHz, Chloroform-d) δ 9.93 (d, J = 8.3 Hz, 1H), 5.90 (m, 1H), 5.16 – 5.05 (m, 2H), 2.61 (m, 2H), 2.23 (m, 2H), 2.07 (m, 2H), 2.02 – 1.96 (m, 5H), 1.69 (m, 3H), 1.62 (m, 6H).
